# Supplementary material for: Metabolomics Analysis Provides Novel Insights into the Difference in Meat Quality between Different Pig Breeds
Source: Foods. 2023 Sep 19;12(18):3476. doi: 10.3390/foods12183476 (PMC10528157; doi:10.3390/foods12183476)
Supplement: Supplementary file 1 [file foods-12-03476-s001.zip › foods-2585454-supplementary.pdf]

Table S1. Feed ingredients and nutrient levels of the basal diet.

Ingredient and nutritive level	60-90 kg	90 kg-slaughter
Ingredients (%)		
Corn	78.08	79.80
Soybean meal	17.50	13.00
Wheat bran	1.80	4.60
L-Lysine-HCl	0.38	0.37
DL-Methionine	0.08	0.05
L-Threonine	0.13	0.12
L-Tryptophan	0.01	0.02
Choline chloride	0.06	0.06
Limestone	0.74	0.76
$CaHPO_4$	0.42	0.42
NaCl	0.40	0.40
Premix[1]	0.40	0.40
Total	100.00	100.00
Nutrient levels (%)		
Digestible energy, MJ/kg	13.80	13.68
Crude protein	14.00	13.00
Calcium	0.55	0.55
Total phosphorus	0.53	0.53
Available phosphorus	0.30	0.30
Digestible Lys	0.87	0.77
Digestible Met + Cys	0.51	0.45
Digestible Thr	0.57	0.50
Digestible Trp	0.15	0.13

[1] Premix provides per kg of basal diet: Vitamin A, 5100 IU; Vitamin D_3, 2670 IU; Vitamin E, 40 IU; Vitamin K_3, 2.8 mg; Vitamin B_1, 2 mg; Vitamin B_{12}, 0.04 mg; Vitamin B_6, 5.5 mg; Vitamin B_2, 6.4 mg; Vitamin B_5, 21.5 mg; Folic acid, 1 mg; Biotin, 0.2 mg; Nicotinamide, 40 mg; Fe, 110 mg; Cu, 16 mg; Mn, 26 mg; Zn, 34 mg; I, 0.7 mg; Se, 0.4 mg.

Table S2. Primers used in RT-qPCR.

Genes	Sequence	GenBank accession no.
β-actin	F:5′-CCACGAAACTACCTTCAACTCC-3′	DQ845171
	R:5′-GTGATCTCCTTCTGCATCCTGT-3′	
MyHC I	F:5′-GAAGCGCAATGTTGAAACCG-3′	NM_001104951
	R:5′-AACCTCCCGACTCTTGACCC-3′	
MyHC IIa	F:5′-CATTGAGGCCCAGAATAGGC-3′	NM_214136
	R:5′-TGCTTCCGTCTTCACTGTCAC-3′	
MyHC IIb	F:5′-GACTCTGGCTTTCCTCTTTGC-3′	NM_001123141
	R:5′-GAGCTGACACGGTCTGGAAA-3′	
MyHC IIx	F:5′-TTGACTGGGCTGCCATCAAT-3′	NM_001104951
	R:5′-GCCTCAATGCGCTCCTTTTC-3′	
AMPKα1	F:5′-GGTGTAAGGAAAGCAAAATGGC-3′	NM_001167633
	R:5′-TGTGGAGTAGCAGTCCCTGATTT-3′	
PPARδ	F:5′-AGGCATCAGGCTTCCACTACG-3′	NM_001130241
	R:5′-ACCTGCGGGTTGTGCTGACT-3′	
MEF2C	F:5′-CAGTCTCCATCCCAGTGTCCAG-3′	NM_001044540
	R:5′-GTTACCAGGTGAGACCAGCAGA-3′	
FoxO1	F:5′-GGTCAAGAGCGTGCCCTACT-3′	NM_214014
	R:5′-GAGCATCCACCAGGAACTTTTT-3′	
TBX15	F:5′-GGAGATACTTGGATGAGACAGGTG-3′	XM_001926903
	R:5′-TGCTGGTTCTGATAGGCTGTGA-3′	
NFATC1	F:5′-CCTCGCATTGAGATCACATCCT-3′	NM_214161
	R:5′-TTGCAGCTACGGGACGACA-3′	
